# Establishment of a Transgenic Mouse Model Specifically Expressing Human Serum Amyloid A in Adipose Tissue

**DOI:** 10.1371/journal.pone.0019609

**Published:** 2011-05-18

**Authors:** Maja Olsson, Sofie Ahlin, Bob Olsson, Per-Arne Svensson, Marcus Ståhlman, Jan Borén, Lena M. S. Carlsson, Kajsa Sjöholm

**Affiliations:** Sahlgrenska Center for Cardiovascular and Metabolic Research, Institute of Medicine at the Sahlgrenska Academy at the University of Gothenburg, Gothenburg, Sweden; Emory University, United States of America

## Abstract

Obesity and obesity co-morbidities are associated with a low grade inflammation and elevated serum levels of acute phase proteins, including serum amyloid A (SAA). In the non-acute phase in humans, adipocytes are major producers of SAA but the function of adipocyte-derived SAA is unknown. To clarify the role of adipocyte-derived SAA, a transgenic mouse model expressing human SAA1 (hSAA) in adipocytes was established. hSAA expression was analysed using real-time PCR analysis. Male animals were challenged with a high fat (HF) diet. Plasma samples were subjected to fast protein liquid chromatography (FPLC) separation. hSAA, cholesterol and triglyceride content were measured in plasma and in FPLC fractions. Real-time PCR analysis confirmed an adipose tissue-specific hSAA gene expression. Moreover, the hSAA gene expression was not influenced by HF diet. However, hSAA plasma levels in HF fed animals (37.7±4.0 µg/mL, n = 7) were increased compared to those in normal chow fed animals (4.8±0.5 µg/mL, n = 10; p<0.001), and plasma levels in the two groups were in the same ranges as in obese and lean human subjects, respectively. In FPLC separated plasma samples, the concentration of hSAA peaked in high-density lipoprotein (HDL) containing fractions. In addition, cholesterol distribution over the different lipoprotein subfractions as assessed by FPLC analysis was similar within the two experimental groups. The established transgenic mouse model demonstrates that adipose tissue produced hSAA enters the circulation, resulting in elevated plasma levels of hSAA. This new model will enable further studies of metabolic effects of adipose tissue-derived SAA.

## Introduction

Obesity, and especially central obesity, is a major risk factor for cardiovascular disease [1]. The adipose tissue produces a variety of adipokines that act both locally within the adipose tissue and systemically when released into the circulation. Obesity is associated with a low grade inflammation with slightly elevated serum levels of acute phase proteins including C-reactive protein (CRP) and serum amyloid A (SAA) [2], [3]. Elevated serum levels of SAA are associated with insulin resistance, type 2 diabetes and may have a prognostic value for cardiovascular disease [4], [5], [6], [7], [8].

In the acute phase response, SAA is produced by the liver and its serum levels can rise thousand-fold, but the function of SAA is poorly understood. Extrahepatic production of SAA has been found [9], and we, and others, have previously reported that in the non acute phase, adipocytes are the main producers of SAA in obese subjects [10], [11]. SAA gene expression is increased in human hypertrophic adipocytes [12], cells that are known to be associated with obesity and insulin resistance [13]. In addition, SAA serum levels are correlated with measures of obesity and reduced during diet-induced weight loss [10]. Furthermore, SAA release from human adipose tissue *in vitro* has been shown to correlate with SAA gene expression [2]. Thus, it is likely that, in humans, the increased fat mass in obesity contributes substantially to SAA levels in the circulation.

Previous studies in humans have shown that SAA may have various effects including promoting proinflammatory cytokine production [2], inducing lipolysis [2], [14], and increasing chemotaxis of inflammatory cells [15], [16]. Furthermore, SAA can remove excess cholesterol from sites of inflammation (reviewed in [17]), and has been suggested to play a role in cholesterol efflux within the adipose tissue [18]. SAA can act as an apolipoprotein, and the majority of SAA in the blood is associated with high-density lipoprotein (HDL). SAA causes displacement of ApoA-I, the predominant HDL apolipoprotein, *in vitro* [19], which may alter HDL properties in a proatherogenic way. Proteoglycans are components of the extracellular matrix and may be important for deleterious lipoprotein retention. SAA contains proteoglycan binding domains [20] and has been suggested to mediate pro-atherosclerotic lipid and lipoprotein retention in the vessel wall. *In vitro*, a higher amount of SAA on the HDL particles increases the binding of HDL to proteoglycans [21] and the idea is further supported by co-localization of SAA with ApoA-I in atherosclerotic lesions in mice [21], [22]. Interestingly, a recent publication showed that SAA, but not CRP, has the ability to directly promote vascular proteoglycan synthesis in a pro-atherogenic manner [23], suggesting that SAA could be a direct link between low-grade inflammation in adipose tissue and cardiovascular disease in obese subjects.

The SAA protein is transcribed from the two homologous genes SAA1/SAA2 in humans, and from Saa1/Saa2 in mice. However, there are also species differences in the SAA gene family. In mice, Saa3 is extrahepatically expressed, while SAA3 in humans is a pseudo gene. Hence, in mice, the Saa1/Saa2 and Saa3 genes are expressed in adipose tissue whereas in humans only the SAA1/SAA2 genes are transcribed. It has also been shown that, only the expression of Saa3 is increased after HF diet in mice [24]. Even though obese mice also display increased SAA levels in serum, absence of SAA3 in the serum was recently reported [25], indicating that in obese mice, SAA in the circulation has a hepatic origin.

The mature protein encoded by SAA1/SAA2 is highly conserved between mouse and human [26]. In contrast, the mouse SAA1/SAA2 and SAA3 proteins are distinct proteins with a sequence homology of only 63–65% [26]. Because of these species differences over expression of human SAA1 is likely to be the most suitable model when addressing questions related to adipose tissue expression of SAA in human obesity. Others have studied the effects of the human SAA protein (hSAA) in mice using adenoviral over expression [23], [27], [28], [29]. However, this method only results in a short-term hSAA production from the liver. Thus, these studies have not mimicked the chronic increase in adipose tissue-derived SAA in obese humans. We have therefore developed a mouse model where hSAA is over expressed in adipose tissue to mimic the situation in human obesity. Consequently, this model enables studies of the systemic and local effects of adipocyte-derived SAA and provides a tool that can be used to define the role of adipose tissue derived SAA in the development of metabolic disease.

## Methods

### Ethics statement

All manipulations involving mice were performed using protocols approved by the local Ethics Committee for Animal Studies at the Administrative Court of Appeals in Gothenburg, Sweden, approval numbers 201-2006 and 281-2008.

### Animals

**Figure 1:**
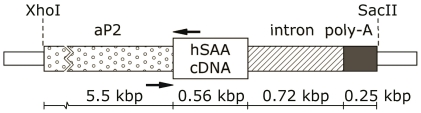
Generation of transgenic mice overexpressing hSAA in adipose tissue. A schematic representation of the aP2 promoter-hSAA fusion gene. Human SAA1 cDNA was ligated to the aP2 promoter/enhancer as described in “Methods”. *Dotted* box aP2 promoter, *hatched* box, rabbit β-globin intron, *grey* box, polyadenylation signal. Restriction enzyme digestion with XhoI and SacII enzymes generated fragments of 7.2 kilobases that were used in pronuclei injections. Locations of genotyping primers are indicated with arrows.

Transgenic founder mice were generated on a C57BL/6J background. The cDNA from human SAA1 (hSAA) was cloned into the pCR 2.1 vector (Invitrogen, Carlsbad, CA) and verified by sequencing. A SmaI-EcoRV digested fragment containing hSAA1 was ligated into Sma1 linearized pBluescript SK II+ vector (Stratagene, La Jolla, CA) downstream of the fatty acid binding protein 4 (aP2) promoter (kindly provided by Dr. Bruce Spiegelman) [30]. A SmaI-BamHI fragment containing the SV40 polyadenylation sequence from the pSI vector (Promega, Madison, WI) was ligated into BamHI-SmaI linearized pBS SK+ vector. A Sma1-HincII fragment containing aP2-hSAA1 was inserted into EcoRV linearized pBluescript SK+ (Stratagene, La Jolla, CA) upstream of the SV40 polyadenylation sequence. Finally, a BamHI-BglII blunted rabbit β-globin gene intron was inserted into the SmaI site, downstream of aP2-hSAA and upstream of the polyadenylation site. A 7190 bp SacII-XhoI fragment (Figure 1) was microinjected into C57BL/6J fertilized eggs using pronuclei injection and eggs were implanted in the oviduct of pseudopregnant recipients. All restriction enzymes were purchased from New England Biolabs (Ipswich, MA). Transgenic mice were identified by PCR with forward and reverse primers 5′-CCAGGGAGAACCAAAGTTGA and 5′-CCCGAGCATGGAAGTATTTG, respectively. The endogenous β-globin gene was co-amplified as an internal control.

Subsequent breeding was done against C57BL/6J mice (Charles River, Sulzfeld, Germany) to generate heterozygous SAA-transgenic mice. Male animals of F2 or later generations were used in experiments. Animals were weaned at 3 weeks of age and housed 3–6 per cage in a temperature-controlled (25°C) facility with a 12-h light-dark cycle with free access to chow and water. From the age of 13 weeks age-matched wild type and hSAA transgenic animals were fed either a normal chow (NC) or a pelleted high fat (HF) diet (60 kcal% fat; D12492, Research Diets, New Brunswick, NJ). At the end of the experiment mice were sacrificed after a 4-hour fast. Using heart puncture blood was collected in potassium EDTA tubes (Sarstedt, Nümbrecht, Germany) from Isofluran (Baxter, Kista, Sweden) inhalation anesthetised animals. Brown adipose tissue (BAT), epididymal adipose tissue (eWAT), retroperitoneal adipose tissue (rWAT), gastrocnemius muscle, heart, liver, kidneys and spleen, were dissected, weighed, snap frozen in liquid nitrogen and stored at −80°C until RNA extraction.

### Body composition

Total body weight was recorded at 11 weeks of age and weekly during the HF or corresponding NC feeding period. Analysis of total body fat was performed by dual energy X-ray absorptiometry (DEXA) using the Lunar PIXImus II Densitometer with version 2.10.041 software (GE Healthcare, Waukesha, WI) after 17 weeks of HF or NC diet. During DEXA analysis, mice were anesthetized with Isofluran.

### RNA preparation and gene expression analysis

RNA from the dissected tissues was prepared using the RNeasy lipid mini kit with TissueLyser homogenization (Qiagen, Chatsworth, CA) and the RNA was then reverse transcribed using the High Capacity cDNA RT kit (Applied Biosystems, Foster City, CA). Real-time PCR analysis was performed using TaqMan Gene Expression Master Mix and cDNA corresponding to 10 ng of RNA per reaction with an ABI Prism 7900HT (Applied Biosystems). The following TaqMan gene expression assays were used; Hs00761940_s1 (human SAA), Mm00441203_m1 (mouse Saa3), and Mm00445878_m1 (mouse Fabp4). The Mm00725448_s1 assay was used for detection of the endogenous reference transcript ribosomal protein, large, P0 (Rplp0; all reagents were from Applied Biosystems). A standard curve was analysed for each assay with a serial dilution of cDNA synthesized from pooled RNA. Samples and standards were analysed in triplicate.

### Blood measurements

After 16 weeks of HF or NC diet, a 4-hour fast preceded blood sampling from the tail vein. Basal glucose and insulin were measured using Accu-Check glucometer (Roche, Stockholm, Sweden) and Ultrasensitive mouse insulin enzyme-linked immunosorbent assay (ELISA) Kit (Chrystal Chem Inc., Downers Grove, IL), respectively. Plasma pools (n = 7–10 animals), from blood collected by heart puncture, were subjected to fast protein liquid chromatography (FPLC) gel filtration on a Superose 6 (10/300GL) column (GE Pharmacia AKTA explorer) [31]. HDL from hSAA-transgenic and wild type mice (n = 6 in each group) was isolated by ultracentrifugation as previously described [32]. Triglycerides and cholesterol were measured in FPLC fractions and in whole plasma using Infinity Cholesterol and Infinity Triglycerides (Triolab AB, Gothenburg, Sweden) with Multiconstituent Calibrator 1E65-04 (Abbott, Solna, Sweden) used as reference. The hSAA protein was measured in plasma from hSAA transgenic mice, in pools of two adjacent FPLC fractions, and in ultracentrifugation samples using a Human SAA ELISA Kit (Biosource, Camarillo, CA). Mouse SAA protein (mSAA) was measured in plasma using the Mouse SAA ELISA Kit (Tridelta Development Ltd., Co. Kildare, Ireland).

### Statistical Analysis

Data is reported as mean ± standard deviation (SD) unless indicated otherwise. Statistical analysis was performed using SPSS (version 16.0; SPSS; Chicago, IL, USA). Differences between groups were assessed with the Mann-Whitney U-test. Differences in growth curves were assessed with repeated measures analysis of variance (ANOVA). Nonparametric correlations were performed using Spearman rank correlation test. A p-value less than 0.05 was regarded statistically significant.

## Results

### Analysis of mRNA expression in mice overexpressing hSAA in adipose tissue

**Figure 2:**
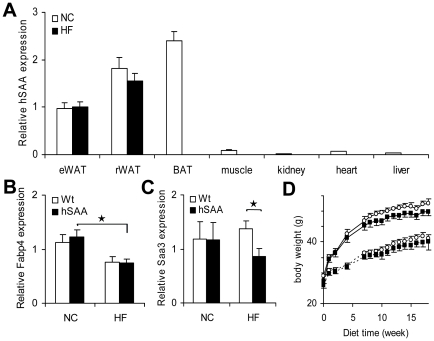
Analyses in hSAA transgenic male mice: gene expression, and animal growth. (**A**) hSAA gene expression in various tissues including epididymal white adipose tissue (eWAT), retroperitoneal white adipose tissue (rWAT) and brown adipose tissue (BAT) was measured in hSAA transgenic animals (n = 7) fed NC diet. hSAA gene expression was also measured in eWAT and rWAT from hSAA mice (n = 10) after 18 weeks of HF diet. Expression was normalized to Rplp0. (**B**) and (**C**) Gene expression of Fabp4 and Saa3 in eWAT (n = 7–10 male animals in each group), respectively. Expression was normalized to Rplp0. (**D**) Growth curves of wild type and hSAA transgenic animals fed NC (dashed line) or HF (solid line) diet for 18 weeks. Open circles, wild type mice; filled squares hSAA mice (n = 7–10 per group).*, p<0.05. Values are given as mean ± SEM.

A construct containing the mouse aP2 promoter/enhancer region was used to generate a mouse model with adipose tissue specific expression of hSAA (Figure 1). Three transgenic founders were obtained from pronuclei injections and one transgenic strain was established. Transgenic animals were viable, produced normal litter sizes and had no visible phenotype. Real-time PCR analysis confirmed that the transgenic construct was specifically expressed in adipose tissue (Figure 2A), and that the hSAA transcript was undetectable in wild type animals fed NC or HF diet (n = 4, respectively; data not shown). In muscle and heart, the hSAA expression levels were just above the lower limit of detection and, as expected, hSAA expression was undetectable in tissues from wild type animals. There was no effect of HF feeding on hSAA gene expression in eWAT or in rWAT compared with samples from NC fed animals (Figure 2A). There was no difference in Fabp4 (aP2) expression in transgenic compared to wild type mice (Figure 2B) in eWAT, but the expression was significantly lower in HF compared to NC fed hSAA animals (Figure 2B; p = 0.011). In HF fed animals the expression of Saa3 in eWAT was reduced in hSAA compared with wild type animals (Figure 2C, p = 0.028). However, in animals fed NC the Saa3 expression in adipose tissue was unchanged.

### Analysis of growth and body composition in hSAA transgenic animals

At 11 weeks of age, the body weights of hSAA male mice (25.0±2.1 g, n = 18) were slightly lower compared to wild type male mice (26.3±2.0 g, n = 19; p = 0.046). Groups of animals were challenged with a HF diet resembling a western diet. Even though there was an initial difference in body weight between hSAA and wild type animals, there was no difference in weight gain between hSAA and wild type mice fed NC or when challenged with HF diet (Figure 2D).

Total body fat content was measured with DEXA after 17 weeks of either NC or HF diet. Total body fat was similar in hSAA and wild type male mice on the same diet (data not shown). In line with this finding, there were no major discrepancies in adipose tissue depot sizes (Table 1). At the end of the study, hSAA animals fed a NC diet had similar adipose tissue depot sizes as wild type animals both in absolute values and when related to body weight (Table 1). Transgenic hSAA HF fed animals displayed significantly increased eWAT depot size both in absolute mass (p = 0.019) and when expressed as percentage of body weight (p = 0.034) compared to wild type mice fed a HF diet. The same animals displayed no change in rWAT or BAT mass.

**Table 1:** Body weight and adipose tissue depots.

	NC	Absolute weight (mg)	Relative weight (% of body weight)	HF	Absolute weight (mg)	Relative weight (% of body weight)
Body wt (g)	hSAA	40.0±4.3	-	hSAA	49.8±4.3	-
	Wt	41.2±3.1	-	Wt	52.5±3.8	-
BAT	hSAA	100±19	0.25±0.04	hSAA	133±44	0.26±0.08
	Wt	117±23	0.28±0.05	Wt	151±29	0.29±0.05
eWAT	hSAA	1837±298	4.59±0.48	hSAA	1873±438*	3.84±1.18*
	Wt	1859±266	4.51±0.47	Wt	1509±183	2.89±0.43
rWAT	hSAA	544±64	1.37±0.19	hSAA	717±113	1.45±0.27
	Wt	547±55	1.33±0.16	Wt	688±139	1.31±0.24

Values are presented as mean±SD. Body wt, body weight; NC, normal chow; HF, high fat; BAT, brown adipose tissue depot; eWAT, epididymal white adipose tissue depots; rWAT, retroperitoneal white adipose tissue depots; hSAA, hSAA male animals; Wt, wild type male animals. n(NC, hSAA) = 7, n(NC, Wt) = 8, n(HF, hSAA) = 10 and n(HF, Wt) = 10.

*p<0.05 between hSAA and Wt animals.

Transgenic hSAA animals fed HF diet had reduced absolute weight of kidneys and heart (p = 0.023 and p = 0.049, respectively). However, when relating to body weight, these significances were lost. The weight of kidneys and heart from animals fed normal chow was similar between wild type and hSAA animals. Furthermore, hSAA animals on HF, but not on NC, had significantly reduced spleen weight compared to wild type mice both in absolute mass and in proportion to body weight (p = 0.001 for both tests). The weights of gastrocnemius muscle, liver or brain were similar in wild type and hSAA animals fed NC or a HF diet (data not shown).

### Analysis of plasma levels of SAA and markers of metabolic status

**Figure 3:**
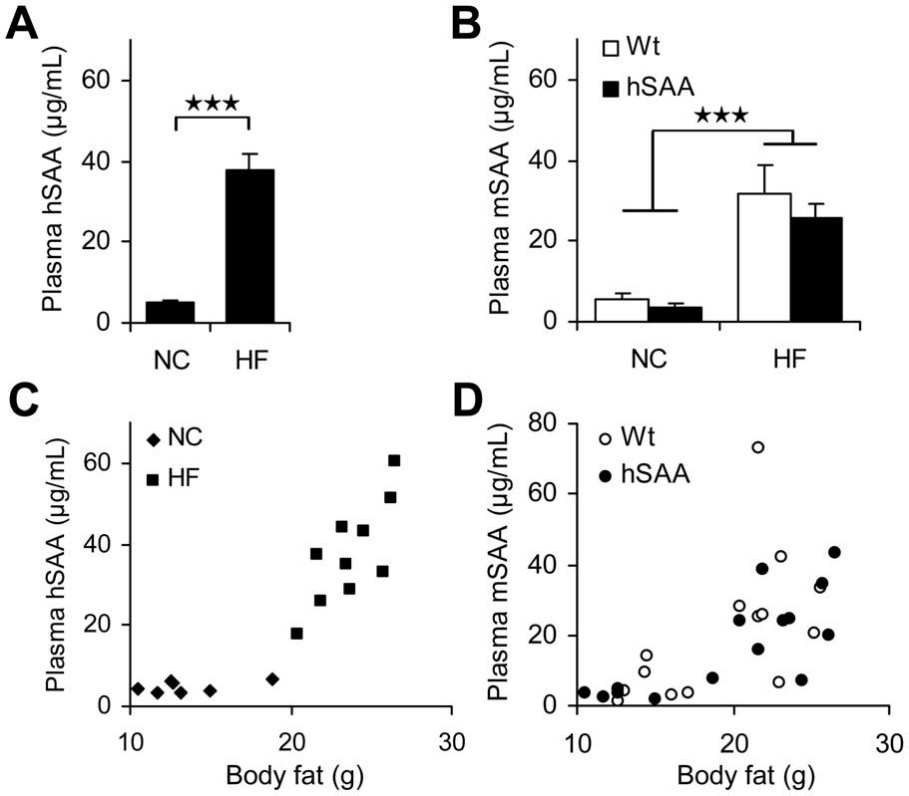
Analyses of SAA plasma levels. (**A**) Plasma levels of hSAA in NC (n = 7) compared to HF (n = 10) fed male animals. (**B**) Plasma levels of mSAA in NC (wild type (Wt) and hSAA; n = 7 and n = 6, respectively) and HF fed (Wt and hSAA; n = 8 and n = 9, respectively) animals. (**C**) Plasma levels of hSAA in relation to body fat quantified using DEXA analysis in transgenic male animals. (**D**) Plasma levels of mSAA in relation to body fat quantified using DEXA analysis in wild type (n = 15) and hSAA (n = 15) transgenic male animals.***, p<0.001. Values are given as mean ± SEM.

An ELISA specific for human SAA was used to analyse hSAA concentrations in plasma from hSAA transgenic animals. We found that the hSAA protein was present in plasma from both NC (n = 7) and HF (n = 10) fed hSAA animals, while hSAA was undetectable in plasma from wild type animals (n = 8). Furthermore, significantly increased levels of hSAA were found in plasma from HF fed compared to NC fed hSAA animals (p = 0.0001, Figure 3A). The plasma concentrations of mouse SAA were measured with an ELISA that showed no cross reactivity towards the hSAA protein (data not shown). The concentration of mSAA in three plasma samples (from one hSAA and two wild type mice) from HF fed animals were above detection limit of the assay. These samples were omitted from mSAA analyses. Plasma levels of mSAA were increased in HF compared to NC fed animals (p<0.0001; Figure 3B). We found no difference in plasma mSAA levels between hSAA and wild type mice (Figure 3B). Total amounts of plasma cholesterol and triglycerides were also measured in individual plasma samples but the levels were unaltered between hSAA and wild type animals within both the NC fed group (cholesterol: 3.0±0.2 and 3.0±0.4 mmol/L; triglycerides: 0.7±0.08 and 0.7±0.09 mmol/L in wild types and hSAA mice, respectively) and the HF fed group (cholesterol: 5.5±0.9 and 5.3±1.0 mmol/L; triglycerides: 0.52±0.13 and 0.55±0.09 mmol/L in wild types and hSAA mice, respectively). Blood glucose and insulin were measured after a 4-hour fast in animals fed 16 weeks HF diet, but neither glucose nor insulin levels differed between wild type and hSAA animals (glucose: 12.9±2.3 and 12.3±2.4 mmol/L; insulin: 2.7±1.0 and 2.0±0.8 ng/mL in wild types and hSAA mice, respectively).

### Plasma levels of SAA in relation to body composition and markers of metabolic status

In humans, SAA plasma levels are correlated with the amount of body fat [10]. Therefore, we wanted to test if the amount of adipose tissue was associated with plasma hSAA levels in hSAA animals. Figure 3C shows that hSAA plasma levels in hSAA animals fed NC and HF diet correlate with the total amount of body fat measured by DEXA analysis (r = 0.88, p = 4*10^−6^). When the results were analysed separately for animals given NC and HF diets, the correlation remained significant (r = 0.65, p = 0.043) in animals challenged with HF diet only. Plasma mSAA levels correlated with body weight (r = 0.79 p = 1.8*10^−7^), and total amount of body fat (r = 0.71 p = 1.0*10^−5^; Figure 3D).

The relationships between plasma hSAA and total amount of cholesterol, total amount of triglycerides, body weight, and adipose tissue hSAA expression were also analysed. Plasma levels of hSAA correlated with body weight and plasma levels of total cholesterol when analysing NC and HF fed animals together (Table 2). However, both correlations were lost when analysing HF and NC fed animals separately (Table 2). Neither hSAA gene expression in eWAT nor in rWAT correlated to plasma hSAA levels (Table 2). The hSAA gene expression in BAT was analysed in NC fed animals only and correlated with plasma hSAA levels (Table 2).

**Table 2:** Spearman correlation coefficients (r) between hSAA plasma levels, body weight, and plasma lipids in hSAA male animals.

	r(NC+HF)	p	r(NC)	p	r(HF)	p
Body weight (g)	0.79	0.00043	0.64	n.s.	0.19	n.s.
Plasma cholesterol (mmol/L)	0.77	0.0003	0.50	n.s.	0.08	n.s.
Plasma triglycerides (mmol/L)	−0.35	n.s.	0.64	n.s.	0.06	n.s.
eWAT hSAA expression	0.24	n.s.	0	n.s.	0.44	n.s.
rWAT hSAA expression	−0.3	n.s.	0.82	n.s.	-0.16	n.s.
BAT	-	-	0.79	0.036	-	-

Values are presented as mean±SD. n(NC+HF) = 17, n(NC) = 7, n(HF) = 10, eWAT, epididymal white adipose tissue; rWAT, retroperitoneal white adipose tissue; BAT, brown adipose tissue.

*** p<0.001, * p<0.05, n.s. not significant.

### Lipoprotein profiles and distribution of hSAA in plasma from transgenic mice

**Figure 4:**
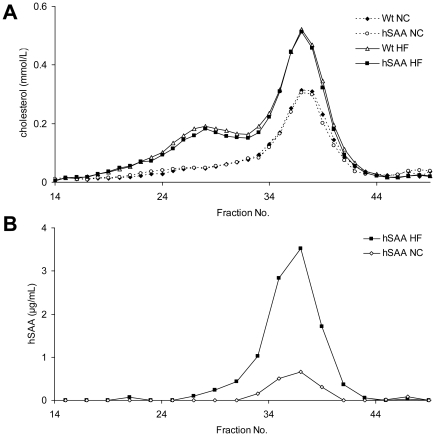
Distribution of cholesterol and hSAA in lipoproteins. Pooled plasma samples from transgenic (hSAA) and wild type (Wt) male animals fed either NC or HF diet (n = 7–10 per group) were subjected to FPLC as detailed in “Methods”. (**A**) Cholesterol levels in plasma FPLC fractions from Wt and hSAA animals. (**B**) hSAA levels in plasma FPLC fractions from hSAA animals.

To investigate whether adipose tissue derived hSAA can modify the composition of lipoproteins, pooled plasma samples (n = 7–10) were subjected to FPLC gel filtration. In response to the 18 week long HF diet, lipoprotein cholesterol content in FPLC fractions increased in a similar way in both wild type and hSAA animals compared to NC fed animals (Figure 4A). Triglyceride distribution among lipoproteins was not changed (data not shown) between wild type and hSAA animals fed NC or a HF diet, respectively. The hSAA concentration, analysed with ELISA in pooled adjacent FPLC-fractions, peaked in HDL containing fractions (Figure 4B) and no hSAA was detected in other fractions. This was also supported by the presence of hSAA in HDL fractions isolated by ultracentrifugation of six individual samples from NC fed hSAA animals (data not shown). Furthermore, hSAA levels in HDL containing FPLC fractions from HF challenged animals were increased compared to the same fractions from animals fed NC diet (Figure 4B).

## Discussion

We here report the establishment and initial characterization of a mouse model with transgenic expression of human acute phase SAA in adipocytes under the control of the aP2 enhancer/promoter. In hSAA transgenic animals, the hSAA protein was present in plasma, demonstrating that adipose tissue-derived SAA is released into the circulation in this model. Furthermore, plasma levels of hSAA in the transgenic mice were similar to those observed in humans and the levels were associated with the amount of total adipose tissue, resembling the association between body fat and serum SAA levels in human subjects. We found no indication of an increased expression of mouse SAA genes in the hSAA transgenic mice. On the contrary hSAA mice subjected to HF diet displayed reduced expression of the mouse Saa3 gene in adipose tissue. Furthermore, plasma levels of mSAA were similar in hSAA and wild type mice and the levels were highly correlated with body fat content as is the case in man [10]. Analyses using both FPLC and ultracentrifugation demonstrated that the hSAA concentration peaked in HDL containing plasma fractions. Hence, this mouse model mirrors the SAA phenotype seen in human obesity and therefore provides a suitable model to determine the direct effects of adipose tissue-derived SAA on the cardiovascular system.

The hSAA transgenic mice displayed no visible phenotype. Although the hSAA mice had slightly lower body weight compared to wild type mice at 11 weeks of age, growth during the 18 subsequent weeks on NC or HF diet did not differ between transgenic and wild type animals. Therefore, we can not rule out that the initially observed difference in weight between wild type and hSAA mice was a spurious finding. hSAA mice challenged with HF diet had reduced spleen sizes, which may indicate an effect on the immune system. Total body fat mass was similar between hSAA and wild type mice, but the epididymal depot mass was decreased after HF diet in wild type animals compared to NC fed mice, and compared to hSAA transgenic mice. This decrease resulted in a significant difference between wild type and transgenic mice. However, if these findings are related to the hSAA over-expression requires further analysis.

The aP2 promoter/enhancer has previously been used in several projects to induce adipocyte specific over-expression [30]. In our study, real-time PCR analysis of hSAA gene expression in various tissues confirmed that hSAA was predominantly expressed in adipose tissue (Figure 2A). In the analysed tissue panel, only very low levels of hSAA expression were detected in muscle and heart. Gene expression of hSAA in three adipose tissue depots revealed different levels of expression: BAT>rWAT>eWAT. This expression pattern has previously been reported in transgenic mice where the aP2 promoter/enhancer was used [33], [34]. It may be speculated that due to the small size of the BAT compared to WAT depots it is possible that the majority of hSAA is produced in WAT in the hSAA transgenic mice. However, this remains to be shown.

Previous studies have shown that SAA serum levels are elevated in obese human subjects and reduced in response to weight loss [2], [10], [35]. Furthermore, we have shown that serum levels of SAA are correlated with the amount of adipose tissue [10]. In mice, it was recently shown that SAA3, the SAA isoform expressed in adipose tissue, does not enter the circulation [25] and that this could be due to SAA3 hyaluron interaction within adipose tissue [36]. In obese human subjects, both the increased SAA expression in adipose tissue and the increase in adipose tissue mass contribute to elevated levels of SAA in serum [2], [10]. In the present study, we have created a model where adipose tissue-derived hSAA does enter the circulation. The plasma levels of hSAA in HF fed mice were increased compared to the levels in the leaner NC fed hSAA mice but the expression levels of hSAA in adipose tissue were unchanged suggesting that the high hSAA levels in plasma are due to the increased adipose tissue mass. This is further supported by the high correlation between plasma levels of hSAA and amount of body fat. In line with previous studies with long-term HF feeding [37], [38], [39] we found a decreased gene activity of the aP2/Fabp4 gene in epididymal adipose tissue from HF fed mice. A reduced induction of the aP2 promoter may explain why the level of hSAA expression was not increased in HF animals.

Importantly, in the established model, plasma hSAA levels in transgenic mice fed HF and NC were in the same ranges as in obese and lean human subjects [2], [10], supporting the idea that this model is suitable for studies aimed at elucidating the effects of adipose tissue derived SAA. Furthermore, this model can be used to clarify the mechanisms for SAA release from adipocytes and macrophages, where the aP2 promoter/enhancer is active [40].

SAA may affect the development of cardiovascular disease through many different mechanisms. For instance, SAA is involved in cholesterol transport, including cholesterol efflux (reviewed in [17]), and a recent report demonstrated that SAA reduces reverse cholesterol transport *in vivo* [41]. In the circulation, SAA is predominantly associated with HDL [42], and SAA-HDL displays potentially reduced antioxidant properties [43] as well as SAA-mediated lipoprotein retention [22]. In the present study, plasma analyses in transgenic mice suggested that hSAA was associated with HDL, indicating that adipose tissue-derived hSAA has similar properties as circulating SAA in man. Furthermore, the presence of hSAA in plasma did not affect the size- or cholesterol-content of the lipoproteins. This is consistent with unaltered lipoprotein profiles in mice with short-term adenoviral overexpression of hSAA [23], [27], and with a recent report of unaltered HDL cholesterol levels in a SAA1 and SAA2 double knock-out mouse model [44]. Wilson *et al.* demonstrated that SAA promotes vascular proteoglycan synthesis *in vitro* and most importantly, that a short-term adenoviral-induced hSAA production was sufficient to obtain pro-atherogenic proteoglycan synthesis *in vivo* [23]. However, atherosclerosis induced by hSAA was not demonstrated [23], possibly due to a rapid decline of adenoviral-induced hSAA plasma levels. In contrast to previous studies of short-term effects of adenoviral-induced liver-produced hSAA, our model enables the study of long-term hSAA effects.

Elevated CRP concentration in the blood is a well recognized risk factor for future cardiovascular disease, although some studies argue against a causative role for CRP [45], [46]. Serum levels of SAA are correlated with levels of CRP and also with cardiovascular risk factors [6] and SAA levels have a prognostic value, in some cases working as a better predictor for clinical outcome than CRP [4], [5]. In mice, CRP levels do not respond to inflammatory stimuli, making mouse models suitable for studying cardiovascular effects of SAA without interference of CRP effects. Furthermore, SAA but not CRP, directly promotes vascular proteoglycan synthesis in a pro-atherogenic manner [23]. In man, SAA serum levels have been reported to be associated with intima-media thickness (IMT), both in chronic disease and in young healthy asymptomatic subjects [47], [48]. Furthermore, SAA is co-localized with apoB in atherosclerotic lesions in HF fed apoE−/− mice [49]. In hyperlipidemic mice, SAA was co-localized with apoA-I [21], [22], apoB [22], and SAA plasma levels were correlated with atherosclerotic lesion area [21]. These data, taken together with the fact that serum levels of SAA are elevated in human obesity, suggest that adipose tissue-derived SAA is an important link between adiposity and the development of cardiovascular disease.

In conclusion, we have established a transgenic mouse model with adipose tissue-specific expression of hSAA under the control of the aP2 promoter/enhancer. The increased adipose tissue-derived hSAA plasma levels in HF fed hSAA mice, correlated with amount of adipose tissue and, in plasma, hSAA concentration peaked in HDL containing fractions. Thus, the established model is similar to the situation found in human obesity and will provide a novel tool for studies of the direct metabolic and vascular effects of adipose tissue-derived hSAA.
